# Patient profiles and P-HEMS specific interventions performed by a HEMS team in the netherlands; A retrospective cohort study of 18,199 patients

**DOI:** 10.1007/s00068-025-02976-7

**Published:** 2025-10-14

**Authors:** Mehdi Badaoui, Niek J. Vianen, Jan C. Van Ditshuizen, Iscander M. Maissan, Robert-Jan Houmes, Michael H.J. Verhofstad, Esther M.M. Van Lieshout, Mark G. Van Vledder

**Affiliations:** 1 Trauma Research Unit Department of Surgery, Erasmus MC, University Medical Center Rotterdam, Rotterdam, The Netherlands; 2 Trauma Centre Southwest Netherlands, University Medical Center Rotterdam, Rotterdam, The Netherlands; 4 Department of Anesthesiology, Erasmus MC, University Medical Center Rotterdam, Rotterdam, The Netherlands; 3 Department of Neonatal and Pediatric Intensive Care, Division of Pediatric Intensive Care, Erasmus MC Sophia Childrens Hospital, University Medical Center Rotterdam, Rotterdam, The Netherlands

**Keywords:** Emergency medical services, Emergency care, Helicopter emergency medical services, Prehospital, Trauma

## Abstract

**Purpose:**

The objective of this study was to provide an overview of dispatches and procedures performed by a Dutch P-HEMS-team during a nine-year period.

**Methods:**

In this retrospective cohort study, all non-cancelled dispatches of the Rotterdam P-HEMS team between January 1, 2015 and December 31, 2023, were analyzed. Dispatches were stratified by specific medical conditions and age. Furthermore, data on P-HEMS specific procedures were collected and temporal trends were analyzed.

**Results:**

During the nine-year study period, the P-HEMS-team was dispatched 36,242 times, of which 18,396 (50.8%) dispatches were cancelled before arrival on scene. In 17,846 (49.2%) non-cancelled dispatches, the P-HEMS team assessed 18,199 patients. The main reasons for dispatch were trauma (62.9% in adults, 41.5% in children), out of hospital cardiac arrest (14.8% in adults, 9.8% in children) and neurological disorders (6.8% in adults, 22.3% in children). The number of annual non-cancelled dispatches increased from 1,605 to 2,114 (31.7% increase) during the study period, mostly due to an increase in dispatches for non-traumatic conditions. One or more P-HEMS specific procedures were performed in 7,793 (43.2%) of patients. The most frequently performed procedures were administration of P-HEMS specific medication (*n* = 6,298; 34.9%) and drug assisted advanced airway management (*n* = 5,348; 29.7%).

**Conclusion:**

Over a nine-year period, there has been an increasing demand for P-HEMS care in the Southwestern part of the Netherlands for traumatic and non-traumatic conditions. P-HEMS specific procedures were performed in 43.2% of patients attended by the P-HEMS team. This proportion has remained fairly stable during the study period.

## Introduction

The Netherlands is a northwestern European country of 37,391 km^2^ surface, inhabited by approximately 18 million people in a dominantly urban environment [1, 2]. Physician staffed Helicopter Emergency Medical Services (P-HEMS) in the Netherlands have been providing prehospital emergency medical care since 1995. Currently, four Dutch HEMS-teams are available 24 h per day and can be dispatched by either helicopter or rapid response vehicle throughout the Netherlands to support nurse staffed ground emergency medical services (GEMS) in critically ill and injured patients. The Rotterdam P-HEMS team on duty consists of either an anesthesiologist or a trauma surgeon, a specialized GEMS nurse or emergency department nurse, and a pilot. The physician and nurse are extensively trained for prehospital emergency medicine and can provide additional care to the nurse-staffed GEMS crews in highly complex circumstances and critically ill patients (e.g., drug assisted advanced airway management, administration of specific drugs, or invasive procedures). The four Dutch P-HEMS teams are primarily deployed in designated regions, but can be deployed nationwide if a P-HEMS team in another region has already been deployed or if an incident requires multiple teams [3]. In this way, P-HEMS care should be available nationwide 24/7. Dutch P-HEMS teams are deployed for a variety of conditions, mostly being acute loss of consciousness as a result of major trauma, neurological events, cardiological events, etc [4].

Bi-annual reports by the Dutch Network for Emergency Care (Landelijk Netwerk Acute Zorg) show a gradual nationwide increase in the number of P-HEMS dispatches since 2018 [5]. Unfortunately, these reports do not provide an in-depth analysis of conditions for which Dutch P-HEMS teams are dispatched and if the proportion of certain conditions for which P-HEMS is deployed changes over time. Moreover, the proportion of patients attended by Dutch P-HEMS teams that actually requires P-HEMS specific interventions has not been well documented. A prospective observational study investigating 78 patients reported that HEMS physicians provided additional treatment on top of GEMS treatment in 45% of patients, although the small sample size did not allow for an analysis per specific patient category [6]. This is important, as it may serve to adjust dispatch criteria over time to make sure the right patient receives the right care in the right time.

Therefore, the aim of the current study was to provide a detailed overview of P-HEMS care delivered over an extended period of time and to investigate temporal trends in the type of patients cared for and the type of care delivered.

## Methods

### Study design and setting

This was a retrospective single center cohort study including data of all dispatches where the Rotterdam P-HEMS team arrived on-scene since data was registered online. The Rotterdam P-HEMS serves the southwest region of the Netherlands containing about five million inhabitants living in both densely populated urban areas as well as in rural areas. The study was exempted by the local Medical Research Ethics Committee (MEC-2023-0640).

### Data and text mining

Data on dispatches between January 1, 2015 and December 31, 2023, were retrospectively retrieved from the Rotterdam HEMS registry. This study included only data on P-HEMS deployment and not on inter-hospital transport of patients. Since a substantial amount of data were registered as free text field variables in the registry, data were categorized using excel queries. Data were exported to Microsoft Excel (Microsoft, Version Microsoft 365, 2024, Redmond, USA). For each parameter of interest, queries were developed in Microsoft Excel. Queries consisted of keywords of interest and were specific per variable or category of interest (e.g., cardiac arrest or cardiopulmonary resuscitation (CPR), chest tube or chest drain, but also possible typos or other synonyms). Queries were also made to check for denials in the according free text fields (e.g., no, not, etc.). Conflicting results (e.g., “yes” for a variable of interest and a denial in the same text box) were manually checked by two researchers.

Next, patients were assigned to a specific category (e.g., trauma or emergency medicine) and subcategory (e.g., blunt trauma, penetrating trauma, or burns). Patients who could not be assigned to a category, or in whom the data and text mining assigned them to multiple categories were manually assigned following discussion by two investigators, MB and MGvV. In addition, patients were stratified based on age (adult, 16 years or older; pediatric, 0.2 years to 15 years; and neonatal, < 0.2 years) and by whether or not CPR was performed. Additionally, trend analyses were performed to investigate temporal changes in patient categories.

At last, the proportion of patients who had undergone P-HEMS specific interventions was investigated. P-HEMS specific treatments were categorized as drug assisted advanced airway management, invasive procedures, application of a femoral splint, administration of P-HEMS specific drugs, point-of-care ultrasound (POCUS) examination, and physician assisted ground transportation (P-GT). Since POCUS and P-GT are no interventions in itself, the analysis was performed with and without the inclusion of these procedures. Out-of-hospital cardiac arrest (OHCA) cases were separately analyzed to gain insight in P-HEMS specific interventions for OHCA in adults.

### Data analysis

Data were analyzed using the Statistical Package for the Social Sciences (SPSS) (IBM, SPSS version 28, Illinois, Chicago, USA). Missing values were not imputed. A p-value < 0.05 was considered of statistical significance in all statistical tests, and all tests were two-sided.

Continuous variables, which were all deviated from a Normal distribution according to the Shapiro-Wilk test. Non-normally distributed are presented as median with quartiles (P_25_-P_75_). Categorical variables are presented as number with percentage. For the added value of P-HEMS dispatch including POCUS, including P-GT, and including both, differences in additional value were tested using the McNemar Test. In the time trend analysis, correlation between the procedures and time was tested using Kendall’s tau test.

## Results

### Dispatches

**Fig. 1:**
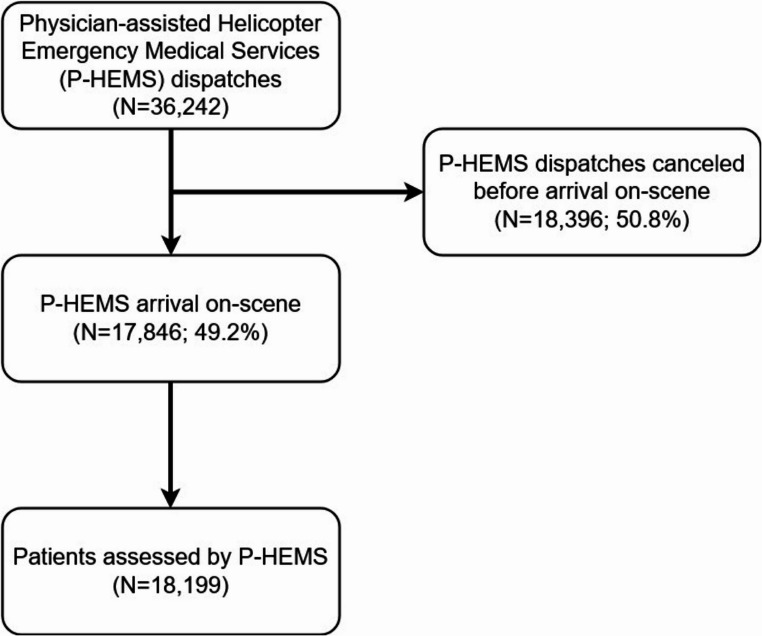
Flowchart of the included P-HEMS-dispatches

During the study period, a total of 36,242 dispatches were registered in the Rotterdam P-HEMS registry (Fig. 1). Of those dispatches, 18,396 (50.8%) were cancelled; in the 17,846 (49.2%) non-cancelled dispatches, 18,199 patients were attended by the P-HEMS team. Of the 17,846 dispatches, there were 13,285 (74.4%) dispatches in which the helicopter was used, in the remaining 4,561 (25.6%) dispatches, the Rapid Response Vehicle was used. Main reasons for dispatch cancellation were: Stable patient according to GEMS team (*n* = 8,938, 48.6%), mechanism of injury not sufficient (*n* = 5,671, 30.1%), patient already deceased (*n* = 1,254, 6.8%), patient already transported by GEMS team (*n* = 927, 5.0%) and other P-HEMS team already on-scene (*n* = 809, 4.4%).

### General patient population, patient categories and age subgroups

Of the 18,199 patients, 14,252 (78.3%) were adult patients (≥ 16 years); 3,326 (18.3%) were pediatric patients (1 month – 16 years), and 450 (2.5%) were neonatal patients (≤ 1 month old). Patients were mostly males (adults *n* = 9,587; 67.4%, pediatric patients *n* = 1,984; 59.7%, neonates *n* = 220; 48.9%). Median age was 48 years for adults (P_25_-P_75_; 30–63 years; Table 1) and 3 years for pediatric patients (P_25_-P_75_; 1.3-8.0 years; Tables 2 and 3).

**Table 1:** Patient categories for non-cancelled HEMS-dispatches in the adult population (16 years or older)

	All Adults (*N* = 14,252)	Adults without CPR (*N* = 10,721)	Adults with CPR (*N* = 3,531)
**Age (year)**	48 (30–63)	46 (29–62)	51 (37–65)
**Male sex**	9,587 (67.4%)	7,093 (66.3%)	2,494 (70.7%)
**Trauma**	**8,962 (62.9%)**	**7,639 (71.3%)**	**1,323 (37.5%)**
Blunt trauma	7,156 (50.2%)	6,326 (59.0%)	830 (23.5%)
* Traffic*	*4*,*258 (29.9%)*	*3*,*765 (35.1%)*	*493 (14.0%)*
* Fall from height*	*2*,*428 (17.0%)*	*2*,*137 (19.9%)*	*291 (8.2%)*
* Other*	*470 (3.3%)*	*424 (4.0%)*	*46 (1.3%)*
Penetrating trauma	934 (6.6%)	791 (7.4%)	143 (4.0%)
* Stab wounds*	*695 (4.9%)*	*606 (5.7%)*	*89 (2.5%)*
* Gunshot wounds*	*220 (1.5%)*	*168 (1.6%)*	*52 (1.5%)*
* Other*	*19 (0.1%)*	*17 (0.2%)*	*2 (0.1%)*
Burns	288 (2.0%)	273 (2.5%)	15 (0.4%)
Electrocution	16 (0.1%)	12 (0.1%)	4 (0.1%)
Strangulation	350 (2.5%)	124 (1.2%)	226 (6.4%)
Drowning	165 (1.2%)	64 (0.6%)	101 (2.9%)
Blast injury/explosion	44 (0.3%)	40 (0.4%)	4 (0.1%)
Other	9 (0.1%)	9 (0.1%)	0 (0.0%)
**Emergency Medicine**	**4,213 (29.6%)**	**2,023 (18.9%)**	**2,190 (62.0%)**
Respiratory	436 (3.1%)	406 (3.8%)	30 (0.8%)
Cardiac	48 (0.3%)	48 (0.4%)	0 (0.0%)
OHCA (etiology not specified)	2,113 (14.8%)	7 (0.1%)	2,106 (59.6%)
Anaphylaxis/allergy	423 (3.0%)	423 (3.9%)	0 (0.0%)
Sepsis	63 (0.4%)	62 (0.6%)	1 (< 0.01%)
Intoxication	339 (2.4%)	325 (3.0%)	14 (0.4%)
CBRN	45 (0.3%)	39 (0.4%)	6 (0.2%)
Bleeding (non-traumatic)	167 (1.2%)	160 (1.5%)	7 (0.2%)
Heat syndromes	25 (0.2%)	21 (0.2%)	4 (0.1%)
Other	554 (3.9%)	532 (5.0%)	22 (0.6%)
**Neurology**	**971 (6.8%)**	**955 (8.9%)**	**16 (0.5%)**
Seizure	378 (2.7%)	373 (3.5%)	5 (0.1%)
Stroke/intracranial hemorrhage	550 (3.9%)	540 (5.0%)	10 (0.3%)
Other	43 (0.3%)	42 (0.4%)	1 (< 0.01%)
**Obstetrics**	**86 (0.6%)**	**84 (0.8%)**	**2 (0.1%)**
HELLP/eclampsia	7 (< 0.01%)	6 (0.1%)	1 (< 0.01%)
Early delivery (< 30w)	28 (0.2%)	28 (0.3%)	0 (0.0%)
Difficult delivery	30 (0.2%)	30 (0.3%)	0 (0.0%)
Post-partum hemorrhage	20 (0.1%)	19 (0.2%)	1 (< 0.01%)
Other	1 (< 0.01%)	1 (< 0.01%)	0 (0.0%)
**Other**	**20 (0.1%)**	**20 (0.2%)**	**0 (0.0%)**
**Prehospital mortality**	**1,627 (11.4%)**	**211 (2.0%)**	**1,416 (40.1%)**

Data are shown as median (P25-P75) or as n (%)

N represents the number of patients for whom data were available

CBRN, Chemical, Biological, Radiological and Nuclear threats; CPR, Cardiopulmonary resuscitation; HELLP, Hemolysis Elevated Liver enzyme levels and Low Platelet levels; OHCA, Out of Hospital Cardiac Arrest

**Table 2:** Patient categories for non-cancelled HEMS-dispatches in the pediatric population (1 month to 16 years)

	All Children (*N* = 3,326)		Children without CPR (*N* = 2,890)	Children with CPR (*N* = 436)
**Age (year)**	3.0 (1.3-8.0)	3.0 (1.4-7.0)		3.0 (1.2-9.0)
**Male sex**	1,984 (59.8%)	1,742 (60.4%)		242 (55.8%)
**Trauma**	**1,379 (41.5%)**	**1,283** **(44.4%)**		**96 (22.0%)**
Blunt trauma	1,068 (32.1%)	1,035 (35.8%)		33 (7.6%)
* Traffic*	*411 (12.4%)*	*390 (13.5%)*		*21 (4.8%)*
* Fall from height*	*588 (17.7%)*	*580 (20.1%)*		*8 (1.8%)*
* Other*	*69 (2.1%)*	*65 (2.2%)*		*4 (0.9%)*
Penetrating trauma	53 (1.6%)	45 (1.6%)		8 (1.8%)
* Stab wounds*	*35 (1.1%)*	*30 (1.0%)*		*5 (1.1%)*
* Gunshot wounds*	*10 (0.3%)*	*7 (0.2%)*		*3 (0.7%)*
* Other*	*8 (0.2%)*	*8 (0.3%)*		*0 (0.0%)*
Burns	165 (5.0%)	165 (5.7%)		0 (0.0%)
Electrocution	3 (0.1%)	3 (0.1%)		0 (0.0%)
Strangulation	24 (0.7%)	6 (0.2%)		18 (4.1%)
Drowning	64 (1.9%)	27 (0.9%)		37 (8.5%)
Blast injury/explosion	2 (0.1%)	2 (0.1%)		0 (0.0%)
Other	0 (0.0%)	0 (0.0%)		0 (0.0%)
**Emergency Medicine**	**1,202 (36.1%)**	**868 (30.0%)**		**334 (76.6%)**
Respiratory	284 (8.5%)	280 (9.7%)		4 (0.9%)
Cardiac	8 (0.2%)	8 (0.3%)		0 (0.0%)
OHCA (etiology not specified)	326 (9.8%)	2 (0.1%)		324 (74.3%)
Anaphylaxis/allergy	174 (5.2%)	174 (6.0%)		0 (0.0%)
Sepsis	82 (2.5%)	82 (2.8%)		0 (0.0%)
Intoxication	31 (0.9%)	30 (1.0%)		1 (0.2%)
CBRN	2 (0.1%)	2 (0.1%)		0 (0.0%)
Bleeding (non-traumatic)	12 (0.4%)	12 (0.4%)		0 (0.0%)
Heat syndromes	3 (0.1%)	3 (0.1%)		0 (0.0%)
Other	280 (8.4%)	275 (9.5%)		5 (1.1%)
**Neurology**	**741 (22.3%)**	**736 (25.5%)**		**5 (1.1%)**
Seizure	706 (21.2%)	701 (24.3%)		5 (1.1%)
Stroke/intracranial hemorrhage	16 (0.5%)	16 (0.6%)		0 (0.0%)
Other	19 (0.6%)	19 (0.7%)		0 (0.0%)
**Other**	**4 (0.1%)**	**3 (0.1%)**		**1 (0.2%)**
**Prehospital mortality**	**141 (4.2%)**	**5 (0.2%)**		**136 (31.2%)**

Data are shown as median (P25-P75) or as n (%).

*N* represents the number of patients for whom data were available.

*CBRN* Chemical Biological Radiological and Nuclear threats, *CPR* Cardiopulmonary resuscitation, *OHCA* Out of Hospital Cardiac Arrest

**Table 3:** Patient categories for non-cancelled HEMS-dispatches in the neonatal population (≤ 1 month)

	All Neonates (*N* = 450)	Neonates with CPR (*N* = 119)	Neonates without CPR (*N* = 331)
**Male sex**	220 (48.9%)	60 (50.4%)	159 (48.0%)
**Neonatal resuscitation**	232 (51.6%)	73 (61.3%)	159 (48.0%)
**Trauma**	31 (6.9%)	2 (1.7%)	29 (8.8%)
**Non-trauma**	187 (41.6%)	44 (37.0%)	143 (43.2%)
**Prehospital mortality**	30 (6.7%)	25 (21.0%)	5 (1.5%)

Data are shown as n (%)

*N* represents the number of patients for whom data were available

*CPR*, Cardiopulmonary resuscitation

In the adult population, the most frequent reason for dispatch was trauma (*n* = 8,962; 62.9%). Of these trauma patients, approximately half of the patients suffered blunt trauma (*n* = 7,156; 50.2%), which were most often road traffic accidents (*n* = 4,258; 29.9%). 1,323 of the 8,962 trauma patients (14.8%) attended by P-HEMS required CPR.

P-HEMS attended 4,231 (29.6%) adult patients with various urgent medical conditions. In this group, OHCA with unspecified etiology was the most common reason for dispatch (*n* = 2,113; 49.9%). Neurological disorders (*n* = 971; 6.8%) and obstetrics (*n* = 86; 0.6%) were relatively infrequent reasons for P-HEMS dispatch in adults. Of the 3,531 adults undergoing CPR, 2,190 (62.0%) patients suffered an urgent medical condition, very often being an OHCA (*n* = 2,106; 96.2%).

Like the adult population, the main dispatch reason for pediatric patients was trauma (*n* = 1,379; 41.5%), mostly blunt (*n* = 1,068; 32.1%). In the pediatric non-trauma group (*n* = 1947; 58,5%), neurological disorders (*n* = 741; 22.3%) OHCA (*n* = 326; 9.8%), respiratory illness (*n* = 284; 8.5%) were the most frequent reasons for P-HEMS non-cancelled dispatches.

The majority of the 450 neonatal patients attended by P-HEMS (*n* = 232, 51.6%) received neonatal care due to poor neonatal transition, within 24 h after delivery.

### P-HEMS specific interventions

P-HEMS specific interventions consisted of drug assisted advanced airway management (*n* = 5,348; 29.7%), invasive procedures (*n* = 767; 4.3%), application of femoral splint (*n* = 69; 0.4%), administration of P-HEMS specific medication (*n* = 6,298; 34.9%), POCUS (*n* = 6,105; 33.9%), and P-GT (*n* = 7,783; 43.7%; Table 4).

**Table 4:** HEMS specific procedures and interventions stratified by age subgroup

	All patients (*N* = 18,028)	Adults (*N* = 14,252)	Children (*N* = 3,326)		Neonates (*N* = 450)
Airway management	5,348 (29.7%)	4,850 (34.0%)	438 (13.2%)	60 (13.3%)	60 (13.3%)
Drug assisted intubation	3,172 (17.6%)	2,913 (20.4%)	242 (7.3%)	17 (3.8%)	
Crash intubation	2,117 (11.7%)	1,880 (13.2%)	194 (5.8%)	43 (9.6%)	
Surgical airway	59 (0.3%)	57 (0.4%)	2 (0.1%)	0 (0.0%)	
**Invasive procedures**	**766 (4.2%)**	**737 (5.2%)**	**26 (0.8%)**	**3 (0.7%)**	
Finger thoracostomy	444 (2.5%)	429 (3.0%)	13 (0.4%)	2 (0.4%)	
Chest tube drainage	171 (0.9%)	162 (1.1%)	8 (0.2%)	1 (0.2%)	
Resuscitative thoracotomy	67 (0.4%)	64 (0.4%)	3 (0.1%)	0 (0.0%)	
Amputation of extremity	1 (< 0.01%)	1 (< 0.01%)	0 (0.0%)	0 (0.0%)	
Emergency caesarean section	4 (< 0.01%)	4 (< 0.01%)	0 (0.0%)	0 (0.0%)	
Extracorporeal membrane oxygenation	71 (0.4%)	69 (0.5%)	2 (0.1%)	0 (0.0%)	
Ultrasound guided puncture (pericardiocenthesis)	18 (0.1%)	18 (0.1%)	0 (0.0%)	0 (0.0%)	
Peripheral nerve block	0 (0.0%)	0 (0.0%)	0 (0.0%)	0 (0.0%)	
**Application of femoral splint**	**69 (0.4%)**	**51 (0.4%)**	**16 (0.5%)**	**0 (0.0%)**	
**P-HEMS specific medication**	**6,298 (34.9%)**	**5,623 (39.5%)**	**646 (19.4%)**	**29 (6.4%)**	
Cardiac/vasopressive medication^a^	2,060 (11.4%)	1,955 (13.7%)	96 (2.9%)	9 (2.0%)	
Adrenalin endotracheal	38 (0.2%)	0 (0.0%)	36 (1.1%)	2 (0.4%)	
Antiarrhythmic agents	931 (5.2%)	881 (6.2%)	48 (1.4%)	2 (0.4%)	
Fibrinogen	148 (0.8%)	143 (1.0%)	4 (0.1%)	1 (0.2%)	
Antibiotics	756 (4.2%)	659 (4.6%)	90 (2.7%)	7 (1.6%)	
Dexamethasone	189 (1.0%)	150 (1.1%)	37 (1.1%)	2 (0.4%)	
Thrombolytics	71 (0.4%)	68 (0.5%)	3 (0.1%)	0 (0.0%)	
Electrolytes^b^	90 (0.5%)	75 (0.5%)	13 (0.4%)	2 (0.4%)	
Antidotes^c^	76 (0.4%)	72 (0.5%)	4 (0.1%)	0 (0.0%)	
Antiemetics	0 (0.0%)	0 (0.0%)	0 (0.0%)	0 (0.0%)	
Hypertonic saline/mannitol	1,621 (9.0%)	1,498 (10.5%)	119 (3.6%)	4 (0.9%)	
Sedatives (incl. midazolam)	3,354 (18.6%)	2,930 (20.6%)	412 (12.4%)	12 (2.7%)	
Muscle relaxants	3,273 (18.2%)	3,001 (21.1%)	255 (7.7%)	17 (3.8%)	
High dose analgesia (exceeding GEMS protocol)	2,036 (11.3%)	1,964 (13.8%)	71 (2.1%)	1 (0.2%)	
**Blood product administration** ^d^	**418 (2.3%)**	**397 (2.8%)**	**20 (0.6%)**	**1 (0.2%)**	
**Ultrasound (POCUS)**	**6,105 (33.9%)**	**5,541 (38.9%)**	**530 (15.9%)**	**34 (7.6%)**	
**Patient transportation**	**7,873 (43.7%)**	**6,465 (45.4%)**	**1,231 (37.0%)**	**177 (39.3%)**	
Helicopter	473 (2.6%)	371 (2.6%)	93 (2.8%)	9 (2.0%)	
P-GT	7,400 (41.0%)	6,094 (42.8%)	1,138 (34.2%)	168 (37.3%)	
**P-HEMS specific procedures**					
Excl. P-GT & POCUS	7,793 (43.2%)	6,895 (48.4%)	828 (24.9%)	70 (15.6%)	
Incl. P-GT	10,064 (55.8%)	8,450 (59.3%)	1,419 (42.7%)	195 (43.3%)	
Incl. POCUS	10,331 (57.3%)	9,133 (64.1%)	1,112 (33.4%)	86 (19.1%)	
Incl. P-GT & POCUS	11,731 (65.1%)	9,932 (69.7%)	1,595 (48.0%)	204 (45.3%)	

Data are shown as n (%)

*N* represents the number of patients for whom data were available

*Numbers in subgroup might not add up to the total number of patients receiving the HEMS specific procedure for the whole group, as patients can receive multiple HEMS specific procedures within the same group*

*P-GT Physician assisted Ground Transportation, POCUS Point Of Care UltraSound Excl., P-GT & POCUS excluding Physician assisted Ground Transport and Point Of Care UltraSound, Incl. P-GT including Physician assisted Ground Transport, Incl. POCUS including Point Of Care UltraSound, Incl. P-GT & POCUS including Physician assisted Ground Transport and Point Of Care UltraSound*

*a e.g. noradrenaline, phenyl, enoximone, dobutamine, adrenaline (in exception: during resuscitation or allergic reactions)*

*b Calcium/Magnesium/NaBic/Potassium*

*c hydroxycobalamine/obidoxim/intralipid*

*d Almost exclusively administration of packed red cells*

Without POCUS and P-GT, P-HEMS specific procedures were performed in 7,794 (43.2%) patients (Table 5). Corresponding proportions were 48.4% (*n* = 6,896) in the adult population, 24.9% (*n* = 828) in the pediatric population, and 15.6% (*n* = 70) in the neonatal population.

**Table 5:** P-HEMS specific procedures stratified by patient category

	All	Trauma	Emerg. Med.^a^	OHCA	Neurology	Obstetrics	Neonatal care	Other
***Adults***	**(N= 14,252)**	**(N= 8,962)**	**(N = 4,213)**	**(N = 2,113)**	**(N = 971)**	**(N = 86)**	**(N = 0)**	**(N = 20)**
Airway management	4,850 (34.0%)	2,331 (26.0%)	2,017 (47.9%)	1,546 (73.2%)	498 (51.3%)	3 (3.5%)	N.A.	1 (5.0%)
Invasive procedures	737 (5.2%)	609 (6.8%)	120 (2.8%)	110 (5.2%)	7 (0.7%)	0 (0.0%)	N.A.	1 (5.0%)
Application of femoral splint	51 (0.4%)	51 (0.6%)	0 (0.0%)	0 (0.0%)	0 (0.0%)	0 (0.0%)	N.A.	0 (0.0%)
Medication	5,623 (39.5%)	3,111 (34.7%)	1,891 (44.9%)	1,182 (55.9%)	604 (62.2%)	13 (15.1%)	N.A.	4 (20.0%)
Blood products	397 (2.8%)	340 (3.8%)	53 (1.3%)	26 (1.2%)	1 (0.1%)	3 (3.5%)	N.A.	0 (0.0%)
POCUS	5,541 (38.9%)	3,845 (42.9%)	1,557 (37.0%)	1,257 (59.5%)	125 (12.9%)	11 (12.8%)	N.A.	3 (15.0%)
Transportation with HEMS Physician (ground & air)	6,465 (45.4%)	3,733 (41.7%)	1,994 (47,3%)	1,152 (54.5%)	685 (70.5%)	48 (55.8%)	N.A.	5 (25.0%)
**P-HEMS specific procedures**								
Excl. P-GT & POCUS	6,895 (48.4%)	3,757 (41.9%)	2,495 (59.2%)	1,722 (81.5%)	624 (64.3%)	15 (17.4%)	N.A.	4 (20.0%)
Incl. P-GT	8,450 (59.3%)	4,859 (54.2%)	2,797 (66.4%)	1,791 (84.8%)	740 (76.2%)	48 (55.8%)	N.A.	6 (30.0%)
Incl. POCUS	9,133 (64.1%)	5,693 (63.5%)	2,765 (65.6%)	1,858 (87.9%)	647 (66.6%)	23 (26.7%)	N.A.	5 (25.0%)
Incl. P-GT & POCUS	9,932 (69.7%)	6,151 (68.6%)	2,978 (70.7%)	1,890 (89.4%)	748 (77.0%)	49 (57.0%)	N.A.	6 (30.0%)
***Children***	**(N = 3,326)**	**(N = 1,379)**	**(N = 1,202)**	**(N = 326)**	**(N = 741)**	**(N = 0)**	**(N = 0)**	**(N = 4)**
Airway management	438 (13.2%)	178 (12.9%)	191 (15.9%)	156 (47.9%)	69 (9.3%)	N.A.	N.A.	0 (0.0%)
Invasive procedures	26 (0.8%)	15 (1.1%)	9 (0.7%)	8 (2.5%)	2 (0.3%)	N.A.	N.A.	0 (0.0%)
Application of femoral splint	16 (0.5%)	12 (0.9%)	3 (0.2%)	0 (0.0%)	1 (0.1%)	N.A.	N.A.	0 (0.0%)
Medication	646 (19.4%)	232 (16.8%)	196 (16.3%)	84 (25.8%)	218 (29.4%)	N.A.	N.A.	0 (0.0%)
Blood products	20 (0.6%)	17 (1.2%)	2 (0.2%)	1 (0.3%)	1 (0.1%)	N.A.	N.A.	0 (0.0%)
POCUS	530 (15.9%)	366 (26.5%)	151 (12.6%)	113 (34.7%)	12 (1.6%)	N.A.	N.A.	1 (25.0%)
Transportation with HEMS Physician (ground & air)	1,231 (37.0%)	507 (36.8%)	363 (30.2%)	137 (42.0%)	361 (48.7%)	N.A.	N.A.	0 (0.0%)
**P-HEMS specific procedures**								
Excl. P-GT & POCUS	828 (24.9%)	300 (21.8%)	302 (25.1%)	182 (55.8%)	226 (30.5%)	N.A.	N.A.	0 (0.0%)
Incl. P-GT	1,419 (42.7%)	562 (40.8%)	575 (39.5%)	213 (65.3%)	382 (51.6%)	N.A.	N.A.	0 (0.0%)
Incl. POCUS	1,112 (33.4%)	530 (38.4%)	348 (29.0%)	200 (61.3%)	233 (31.4%)	N.A.	N.A.	1 (25.0%)
Incl. P-GT & POCUS	1,595 (48.0%)	705 (51.1%)	506 (42.1%)	225 (69.0%)	383 (51.7%)	N.A.	N.A.	1 (25.0%)
***Neonates***	**(N = 450)**	**(N = 0)**	**(N = 0)**	**(N = 0)**	**(N = 0)**	**(N = 0)**	**(N = 450)**	**(N = 0)**
Airway management	60 (13.3%)	N.A.	N.A.	N.A.	N.A.	N.A.	60 (13.3%)	N.A.
Invasive procedure	3 (0.7%)	N.A.	N.A.	N.A.	N.A.	N.A.	3 (0.7%)	N.A.
Application of femoral splint	0 (0.0%)	N.A.	N.A.	N.A.	N.A.	N.A.	0 (0.0%)	N.A.
Medication	29 (6.4%)	N.A.	N.A.	N.A.	N.A.	N.A.	29 (6.4%)	N.A.
Blood products	1 (0.2%)	N.A.	N.A.	N.A.	N.A.	N.A.	1 (0.2%)	N.A.
POCUS	34 (7.6%)	N.A.	N.A.	N.A.	N.A.	N.A.	34 (7.6%)	N.A.
Transportation with HEMS Physician (ground & air)	177 (39.4%)	N.A.	N.A.	N.A.	N.A.	N.A.	177 (39.4%)	N.A.
**P-HEMS** **specific procedures**								
Excl. P-GT & POCUS	70 (15.6%)	N.A.	N.A.	N.A.	N.A.	N.A.	70 (15.6%)	N.A.
Incl. P-GT	195 (43.3%)	N.A.	N.A.	N.A.	N.A.	N.A.	195 (43.3%)	N.A.
Incl. POCUS	86 (19.1%)	N.A.	N.A.	N.A.	N.A.	N.A.	86 (19.1%)	N.A.
Incl. P-GT & POCUS	204 (45.3%)	N.A.	N.A.	N.A.	N.A.	N.A.	204 (45.3%)	N.A.

Data are shown as *n* (%), with percentages representing the proportion within the patient category

*N* represents the number of patients for whom data were available

*POCUS* Point Of Care UltraSound, *HEMS* Helicopter Emergency Medical Services, Excl. *P-GT & POCUS* excluding Physician assisted Ground Transport and Point Of Care UltraSound; Incl. *P-GT* including Physician assisted Ground Transport,Incl. *POCUS* including Point Of Care UltraSound,Incl. *P-GT & POCUS* including Physician assisted Ground Transport and Point Of Care UltraSound, *OHCA* Out of Hospital Cardiac Arrest

*a Including non-traumatic OHCA*

**Fig. 2:**
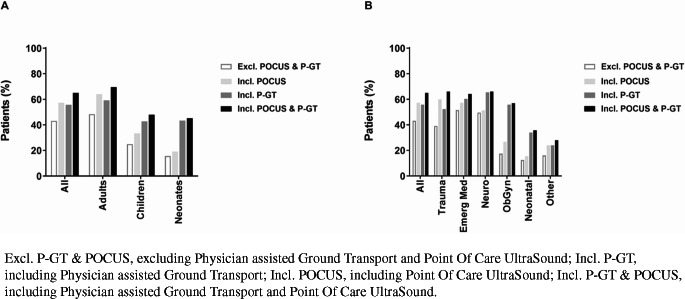
Proportion of non-cancelled P-HEMS dispatches with P-HEMS specific procedures performed, stratified by age subgroup (**A**) and patient category (**B**) Excl. P-GT & POCUS, excluding Physician assisted Ground Transport and Point Of Care UltraSound; Incl. P-GT, including Physician assisted Ground Transport; Incl. POCUS, including Point Of Care UltraSound; Incl. P-GT & POCUS, including Physician assisted Ground Transport and Point Of Care UltraSound

Figure 2 shows the proportion of patients in whom P-HEMS specific procedures were performed, stratified by age subgroup and patient category. When including P-GT, P-HEMS specific procedures were performed in 55.8% of patients (*n* = 10,064). Corresponding proportions in the adult, pediatric, and neonatal populations were 59.3% (*n* = 8,450), 42.7% (*n* = 1,419), and 43.3% (*n* = 195), respectively. Likewise, when including POCUS, the P-HEMS team performed P-HEMS specific procedures in 57.3% of patients (*n* = 10,331). The proportions in the adult, pediatric, and neonatal population for this subgroup were 64.1% (*n* = 9,133), 33.4% (*n* = 1,112), and 19.1% (*n* = 86), respectively. When including both POCUS and P-GT, one or more P-HEMS specific interventions were performed in 65.1% of all patients (*n* = 11,731; Table 4). Proportions were 69.7% (*n* = 9,932) in the adult population, 48.0% (*n* = 1,595) in the pediatric population, and 45.3% (*n* = 204) in the neonatal population.

In the adult population, the P-HEMS team performed P-HEMS specific procedures excluding POCUS and P-GT, in 41.9% (*n* = 3,757) of trauma patients. This was 59.2% (*n* = 2,495) for urgent medical conditions, 64.3% (*n* = 624) for neurology, and 17.4% (*n* = 15) for obstetrics patients, respectively. In the pediatric population, the proportions for trauma are 21.8% (*n* = 300) for non-trauma 55.6% (528), including neurology 30.5% (*n* = 226), and other medical conditions. 25.1% (*n* = 302).

The P-HEMS team performed one or more P-HEMS specific interventions in 1,722 out of 2,113 (81.5%) adults and 182 out of 326 (55.8%) children with non-traumatic OHCA (Table 5).

### Other analyses

Most patients underwent one or two P-HEMS specific procedures (Appendix, Table 6). The highest number of P-HEMS specific procedures performed on a single patient was 11.

**Table 6:** Distribution of the number of HEMS specific procedures

Number of procedures	Excl. P-GT & POCUS	Incl. *P*-GT	Incl. POCUS	Incl. P-GT & POCUS
	(*N* = 18,199)	(*N* = 18,199)	(*N* = 18,199)	(*N* = 18,199)
**Any**	7,793 (43.2%)	10,064 (55.8%)	10,331 (57.3%)	11,731 (65.1%)
**1**	2,784 (15.4%)	3,738 (20.7%)	4,135 (22.9%)	3,933 (21.8%)
**2**	1,920 (10.7%)	1,917 (10.6%)	2,275 (12.6%)	2,496 (13.8%)
**3**	1,412 (7.8%)	1,541 (8.5%)	1,582 (8.8%)	1,794 (10.0%)
**4**	987 (5.5%)	1,353 (7.5%)	1,169 (6.5%)	1,384 (7.7%)
**5**	471 (2.6%)	956 (5.3%)	678 (3.8%)	1,081 (6.0%)
**6**	156 (0.9%)	398 (2.2%)	341 (1.9%)	653 (3.6%)
**7**	50 (0.3%)	119 (0.7%)	105 (0.6%)	271 (1.5%)
**8**	8 (< 0.01%)	31 (0.2%)	33 (0.2%)	84 (0.5%)
**9**	4 (< 0.01%)	7 (< 0.01%)	9 (< 0.01%)	24 (0.1%)
**10**	1 (< 0.01%)	4 (< 0.01%)	3 (< 0.01%)	8 (< 0.01%)
**11**	0 (0.0%)	0 (0.0%)	1 (< 0.01%)	3 (< 0.01%)

Data are shown as n (%)

*N* represent the number of patients for whom data was available

Excl. P-GT & POCUS, excluding Physician assisted Ground Transport and Point Of Care UltraSound; Incl. P-GT, including Physician assisted Ground Transport; Incl. POCUS, including Point Of Care UltraSound; Incl. P-GT & POCUS, including Physician assisted Ground Transport and Point Of Care UltraSound

**Fig. 3:**
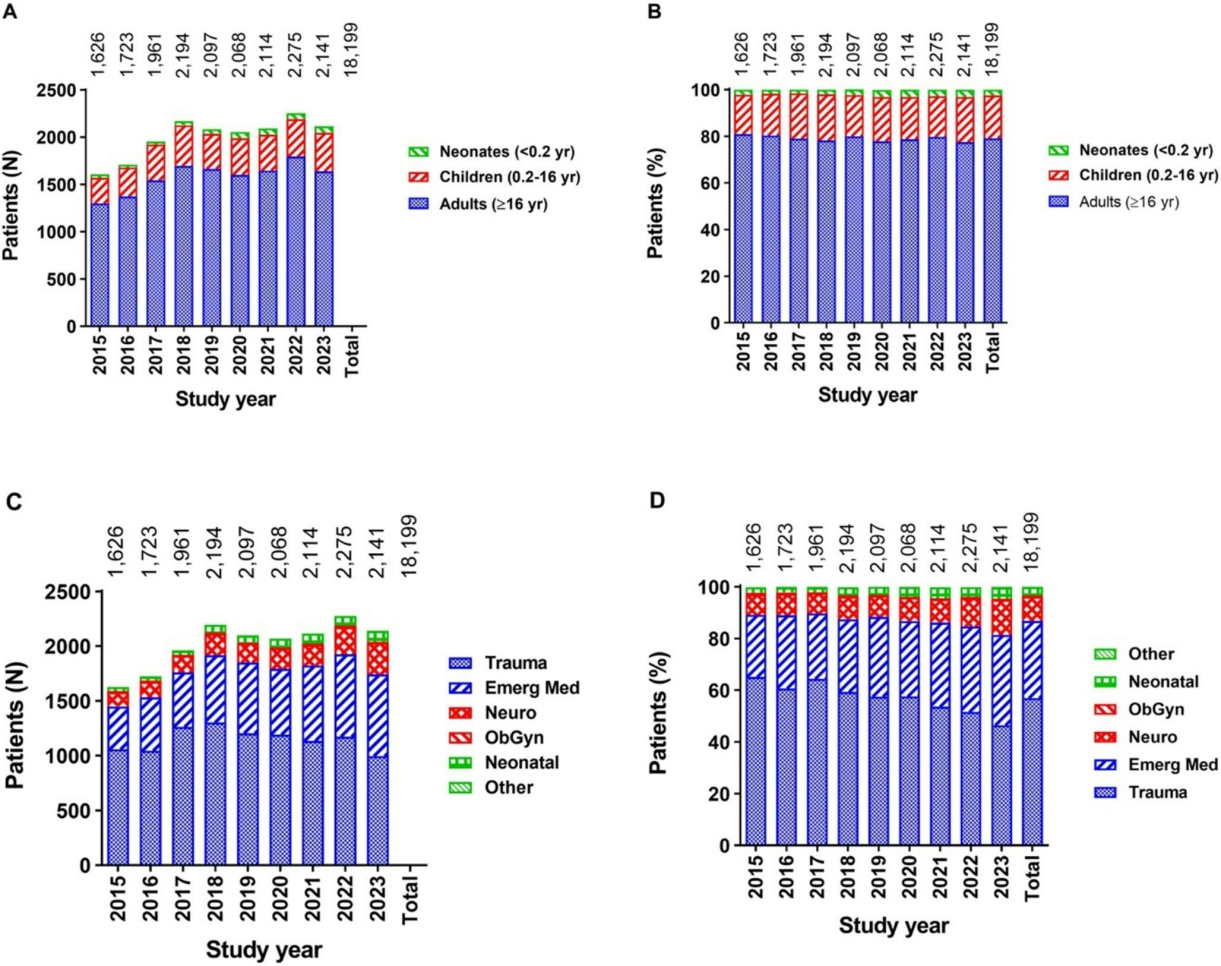
Number (**A** and **C**) and proportion (**B **and **D**) of non-cancelled P-HEMS dispatches stratified by age subgroup (**A** and **B**) or patient category (**B** and **D**)

During the study period, the number of patients assessed by the P-HEMS team on a yearly basis gradually increased from 1,626 to 2,141 patients per year (Fig. 3). The percentual distribution of adults, children, and neonates did not change over time. However, the proportion of trauma dispatches (65.1–46.5%) decreased over time (correlation coefficient *r*=−0,093, *p* < 0.001), while emergency medicine dispatches (24.0–34.9%; *r* = 0.058; *p* < 0.001), neurological disorder dispatches (7.9–13.5%; *r* = 0.044; *p* < 0.001), and neonatal dispatches (2.2–4.4%; *r* = 0.041; *p* < 0.001) increased.

**Fig. 4:**
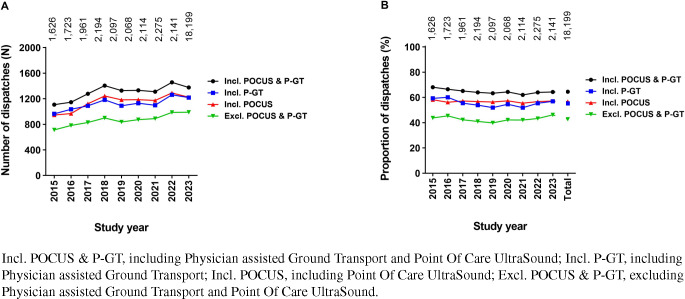
Number (**A**) and proportion (**B**) of noncancelled P-HEMS dispatches with P-HEMS specific procedures performed over time

**Fig. 5:**
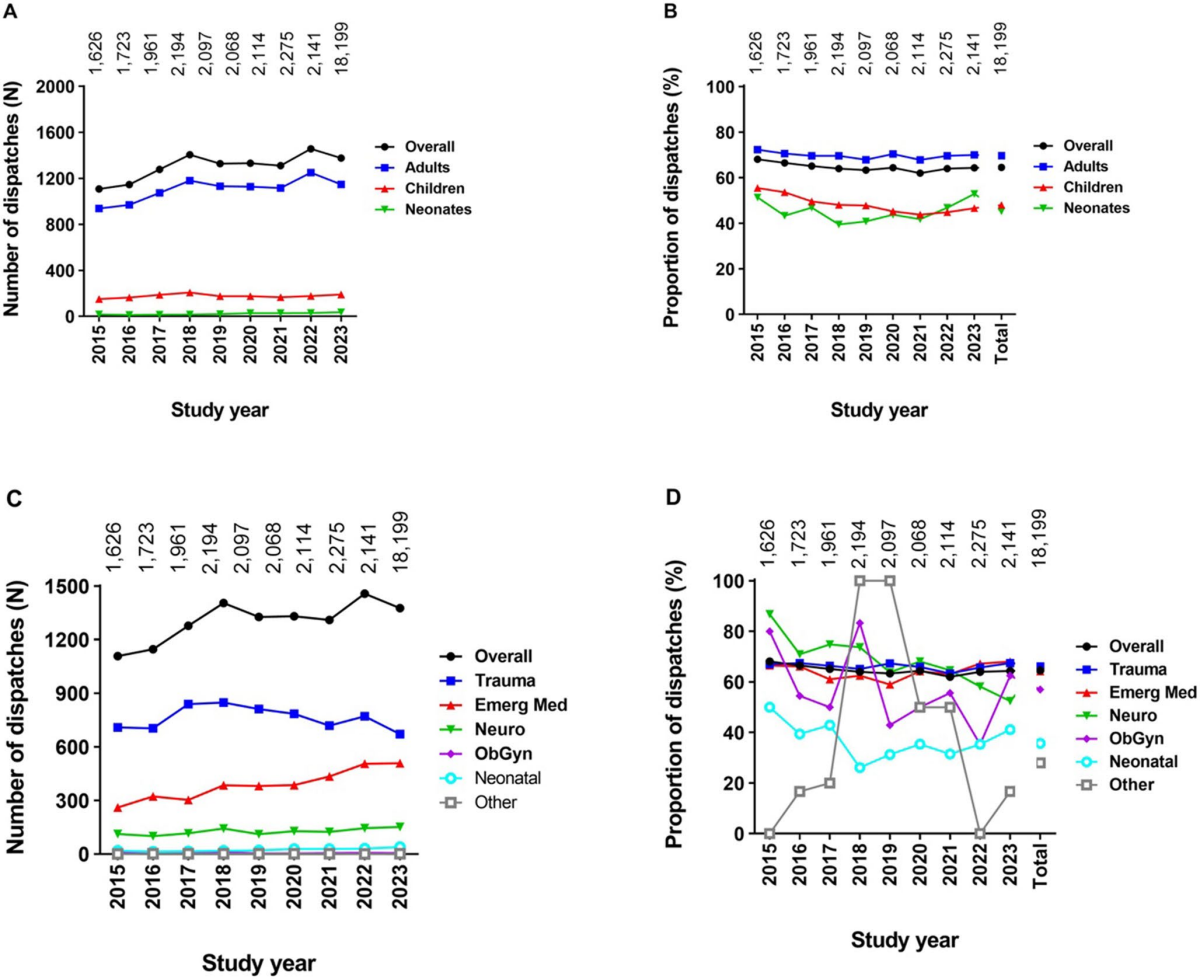
Number (**A** and **C**) and proportion (**B** and **D**) of P-HEMS dispatches with P-HEMS specific procedures over time, stratified by age subgroup (**A** and **B**) or patient category (**C** and **D**)

Both the absolute number of P-HEMS dispatches (31.5% increase during the study period), as well as the absolute number of P-HEMS dispatches in which the P-HEMS team performed one or more P-HEMS specific interventions increased (39% increase during the study period), as well as the proportion of dispatches in which the P-HEMS team performed one or more P-HEMS specific interventions increased from 43.8 to 46.2% (Fig. 4). This positive trend was observed in all age groups and patient categories. Trend analysis of number and proportion of P-HEMS dispatches with P-HEMS specific procedures over time, stratified by age group and type of patient, can be seen in Fig. 5.

## Discussion

In this retrospective cohort study, 18,199 P-HEMS dispatches during a nine-year study period were analyzed and described. P-HEMS specific procedures were performed in 43.2% of patients attended by P-HEMS. Trauma and OHCA were the most common reasons for dispatch in adult and pediatric patients. Both the absolute number of dispatches as well as the proportion of patients undergoing P-HEMS specific interventions increased during the study period.

The results of this study considering the proportion of patients receiving P-HEMS specific interventions are in line with a previous study by Van Schuppen et al. [6] in which the perceived additional value of P-HEMS on scene for GEMS personnel was evaluated. In this study, HEMS physicians provided additional treatment in 45% of assessed patients. In addition, Van Schuppen showed that the presence of the HEMS physician is considered useful by the GEMS personnel when additional treatment is provided, as well as when no additional treatment was provided by the HEMS physician. The presence of the HEMS team was considered useful as they bring diagnostic competencies and clinical decision-making to the scene, give advice and suggestions concerning patient treatment and referral, and simply can serve as additional workforce on scene.

In our study, P-HEMS specific interventions were only performed in 43.2% of patients. However, the true value of a HEMS team on-scene in addition to GEMS teams in critically ill and injured patients may not be completely captured by the interventions performed by P-HEMS alone, as mentioned before. In addition, in many patients in our study, though no P-HEMS specific interventions were registered, some form of POCUS was performed by the HEMS physician on-scene. Our study shows that 33.9% of assessed patients underwent POCUS, which is in line with current literature on P-HEMS operations elsewhere in the world [7, 8]. It was previously shown that POCUS was performed in 34.5% of all prehospital P-HEMS patients and had a therapeutic consequence in 40.7% of patients [9]. In the aforementioned study POCUS seemed to be most effective for patient triage and evaluation of treatment effectiveness in trauma patients. In addition, some patients who did not undergo any P-HEMS specific interventions were accompanied by the HEMS physician during ground transportation to the receiving hospital, suggesting that these patients were deemed to be too unwell to be transported by GEMS personnel alone. When taking POCUS and P-GT into account, the proportion of patients in which any P-HEMS procedure was registered increased to 65.1%.

The proportion of patients in whom P-HEMS specific interventions are registered is substantially lower in pediatric patients when compared to adult patients. This is not surprising, since there is a very low threshold for P-HEMS dispatch for pediatric trauma and other medical pediatric emergencies. Exposure of GEMS teams to severely injured children and children with OHCA is often limited, while this study shows that our service encounters approximately 150 pediatric trauma cases and approximately 35 pediatric OHCA cases annually [10]. This exposure results in rapid accumulation of expertise and experience with these patients per HEMS physician and nurse. Especially P-HEMS dispatches in pediatric (trauma) cases have been extensively studied and found to be positively correlated with additional lives saved [11–17].

Although the absolute number of trauma dispatches remained fairly stable during the study period, there was a gradual decrease of the relative number of trauma dispatches, mostly due to an increase in other patient categories encountered, such as OHCA patients. The proportion of trauma patients in whom P-HEMS specific procedures including P-GT and POCUS were performed remained relatively stable over the years, around 70%. Especially in these patients, the decision to perform any procedure at all can often only be taken once on-scene. While some patients may need resuscitative procedures, such as advanced airway management, on-scene management of chest injuries and/or transfusion of blood products prior to transportation, other patients turn out to be relatively stable and thus require no interventions prior to arrival in the hospital, or have uncompressible bleeding warranting a scoop & run approach. In these patients too, the presence of P-HEMS can be of value in terms of diagnostic capabilities and decision making.

At last, the observed increase in the number of patients with emergency medical conditions may be fully attributed to an increase in the number of dispatches for OHCA. Traditionally, non-traumatic OHCA in adult patients is managed by Advanced Life Support (ALS) certified GEMS teams only. In the Netherlands, P-HEMS are only dispatched in OHCA under special circumstances (pregnancy, suspicion of pulmonary embolism, intoxication, patient age below 30 years, etc.). However, since the start of the On-Scene trial in 2022, OHCA in patients with a suspected age under 50 years has become a standard dispatch criterion for Dutch P-HEMS [11]. While some of these patients may have undergone on-scene extracorporeal CPR (ECPR), many patients that are not eligible for this procedure have had one or multiple P-HEMS specific procedures as part of their CPR procedure or post resuscitation care, suggesting these patients may often benefit from P-HEMS involvement.

The P-HEMS team located near Nijmegen, a more rural part of the Netherlands than our study is focused on, has recently published an article presenting an overview the P-HEMS dispatches for trauma patients, including 2,172 patients [18]. Although their research was solely focused on P-HEMS dispatches on trauma care, it emphasizes the growing interest in a comprehensive overview of Dutch P-HEMS dispatches as well as the limitations of the lack of a joint database for all Dutch P-HEMS teams to facilitate nationwide research on provided value and cost-efficiency. The creation of such a joint database should be a main focus of all Dutch P-HEMS teams [19]. Furthermore, the development of a nationwide (prehospital) electronic health record could also stimulate this type of research and database development, as well as enhance the continuity of care throughout the acute care network regarding handover and efficiency of care.

### Future perspectives

Aforementioned nationwide research could for example be focused on the exact added value regarding mortality and morbidity outcomes of a P-HEMS team when not performing any P-HEMS specific procedures. However, research regarding P-HEMS specific procedures could also be valuable to assess whether these procedures actually impact mortality and morbidity outcomes. In addition, the effectiveness and cost-efficiency of P-HEMS involvement are interesting outcomes to analyze in the especially elderly, considering the current aging in the Dutch population [20]. Furthermore, the cancellation rate of P-HEMS dispatches in our study was 50.8%. This is in line with other studies on the P-HEMS dispatch cancellation rate in the Netherlands [21, 22]. A report from 2014 from the American College of Surgeons states that an overtriage rate up to 35% is considered acceptable for trauma patients, and that an acceptable undertriage rate could be as high as 5% for [23]. This warrants further research in the possibility to sharpen P-HEMS dispatch criteria to minimize overtriage and optimize cost-efficiency overall.

### Limitations

This comprehensive retrospective cohort study has some limitations that should be addressed.

Firstly, this study is of retrospective nature and is therefore inherently at risk of confounding. Furthermore, data about P-HEMS-deployment is usually entered in the registry by the HEMS physician after a dispatch has been concluded. This introduces the possibility of recall bias, as the HEMS physician will face the challenge of remembering all details of a specific dispatch. Also, the registry does not include timing of the performed procedures. It was therefore not possible to discriminate between procedures performed on-scene or during transport. Lastly, because of the data entry in free text fields, the use of text mining queries was necessary. The text mining queries comprised of (parts of) keywords for every variable that could possibly be found in the free text variables. Because of the comprehensiveness of the text mining process, a 100% sensitivity and specificity cannot be guaranteed.

## Conclusion

Over a nine-year period, there has been an increasing demand for P-HEMS care in the Southwestern part of the Netherlands for traumatic and non-traumatic conditions. P-HEMS specific procedures were performed in 43.2% of patients attended by the P-HEMS team. This proportion has remained fairly stable during the study period.

## Data Availability

Data will be made available upon reasonable request to the corresponding author.

## Declarations

### Generative AI in scientific writing statement

The authors did not use a generative artificial intelligence (AI) tool or service to assist with preparation or editing of this work. The author(s) take full responsibility for the content of this publication.
